# Flying-Fox Species Density - A Spatial Risk Factor for Hendra Virus Infection in Horses in Eastern Australia

**DOI:** 10.1371/journal.pone.0099965

**Published:** 2014-06-17

**Authors:** Craig Smith, Chris Skelly, Nina Kung, Billie Roberts, Hume Field

**Affiliations:** 1 Queensland Centre for Emerging Infectious Diseases, Department of Agriculture, Fisheries and Forestry, Brisbane, Queensland, Australia; 2 Biosecurity Intelligence Unit, Department of Agriculture, Fisheries and Forestry, Brisbane, Queensland, Australia; 3 Griffith School of Environment, Griffith University, Brisbane, Queensland, Australia; 4 EcoHealth Alliance, New York, New York, United States of America; 5 GIS People Pty Ltd, Brisbane, Queensland, Australia; Metabiota, United States of America

## Abstract

Hendra virus causes sporadic but typically fatal infection in horses and humans in eastern Australia. Fruit-bats of the genus *Pteropus* (commonly known as flying-foxes) are the natural host of the virus, and the putative source of infection in horses; infected horses are the source of human infection. Effective treatment is lacking in both horses and humans, and notwithstanding the recent availability of a vaccine for horses, exposure risk mitigation remains an important infection control strategy. This study sought to inform risk mitigation by identifying spatial and environmental risk factors for equine infection using multiple analytical approaches to investigate the relationship between plausible variables and reported Hendra virus infection in horses. Spatial autocorrelation (Global Moran’s I) showed significant clustering of equine cases at a distance of 40 km, a distance consistent with the foraging ‘footprint’ of a flying-fox roost, suggesting the latter as a biologically plausible basis for the clustering. Getis-Ord Gi* analysis identified multiple equine infection hot spots along the eastern Australia coast from far north Queensland to central New South Wales, with the largest extending for nearly 300 km from southern Queensland to northern New South Wales. Geographically weighted regression (GWR) showed the density of *P. alecto* and *P. conspicillatus* to have the strongest positive correlation with equine case locations, suggesting these species are more likely a source of infection of Hendra virus for horses than *P. poliocephalus* or *P. scapulatus*. The density of horses, climate variables and vegetation variables were not found to be a significant risk factors, but the residuals from the GWR suggest that additional unidentified risk factors exist at the property level. Further investigations and comparisons between case and control properties are needed to identify these local risk factors.

## Introduction

Hendra virus (genus *Henipavirus*, family *Paramyxoviridae*) was first described in September 1994 in Australia, when it caused an outbreak of typically fatal disease in horses and two close-contact humans [1]–[4]. Sporadic cases continue to occur in horses and humans, with some 80 confirmed or possible equine cases and seven confirmed human cases identified to 31 December, 2012 [5]–[9], and an increase in reported cases since 2006 [10]. Fruit-bats of the genus *Pteropus* (commonly known as flying-foxes) are the natural reservoir of the virus [11]–[16].

Effective treatment for Hendra virus (HeV) infection in horses and humans is lacking, and while a vaccine for horses has been recently released [17], minimising exposure risk remains a fundamental aspect of horse owner management strategies. It is well recognised that ecological processes influence the spatial distribution and patterns of disease risk and disease incidence [18]. Fundamental spatial risk factors comprise the geographic distribution of the pathogen, the natural host and potential spill-over hosts, overlaid by an environmental complexity of ecological and climatic variables that affect the behaviour of the above. The ability of climatic variables to influence host-pathogen interactions and spatial patterns of disease was highlighted in Australia recently with the identification of the role of relative humidity, maximum air temperature and wind speed in the spread of introduced equine influenza virus [19].

A limited number of studies have sought to elaborate the infection and transmission dynamics of Hendra virus in flying-foxes, and identify temporal and spatial risk factors for spill-over of infection from flying-foxes to horses. McFarlane et al (2011) identified a positive association with postcodes containing flying-fox roosts, and with geographic seasonal low rainfall, but found no evident association with horse density and vegetation primary productivity [20]. One of us (Smith, CS., unpublished data) previously found an association with radial proximity to flying-fox roosts, with a statistically significant increased risk of equine cases within 7 km of a known roost. Fogarty *et al* (2008) showed that Hendra virus survival in-vitro decreased with increasing temperature and desiccation [21], supporting the contention that climatic variables likely contribute to infection risk for horses. Correlates with the presence of anti-Hendra virus antibodies in flying-foxes are better understood, and include species, age, location, year, reproductive status (pregnancy or lactation) and season [6], [15], [22], [23].

Reported equine Hendra virus cases to date have been restricted to the adjoining eastern Australia states of Queensland and New South Wales. The limited case numbers prior to 2011 has precluded robust spatial analysis to date, however the unprecedented cluster of 18 separate incidents involving 23 cases in 2011 somewhat relieves this constraint, and with our coincident improved knowledge of horse and flying-fox geographic distribution, invites further spatial analysis. In this paper, we model the spatial occurrence of reported Hendra virus infections in horses, and seek to identify key spatial and environmental risk factors.

## Methods

### Spatial Data

#### Hendra virus equine cases

There were about 80 recorded cases of Hendra infection in horses from 40 discrete case properties in Queensland (n = 31) and New South Wales (n = 9) between September 1994 and 30 December 2012. These cases were reported to and investigated by the Queensland Department of Agriculture, Fisheries and Forestry (DAFF) and the New South Wales Department of Primary Industries (DPI) respectively. The 40 epidemiologically unrelated properties, each with one or more confirmed cases [9], [10] are used in this study to denote individual ‘spill-over events’ of infection from flying-foxes to horses. Case property spatial data in the form of property centroid (latitude and longitude) was sourced in de-identified format from DAFF and DPI.

#### Equine control properties

We randomly selected 1189 control properties from 118,900 registered horse properties in Queensland, New South Wales and Victoria that had not had a confirmed or suspect case of Hendra virus between September 1994 and 31 December 2012. This provided a case-control ratio of nearly 1∶30, and ensured that case properties were matched regardless of variability in property characteristics. Control property spatial data in the form of property centroid (latitude and longitude) was sourced in de-identified format from DAFF, DPI, and the Victorian Department of Primary Industries.

#### Flying-fox occurrence

Flying-fox data are recorded observations of the geographic occurrence of flying-foxes drawn from two sources. Spatial data for the distribution of *P. alecto* and *P. poliocephalus* was derived from Roberts *et al* (2011) [24] and *P. conspicillatus* and *P. scapulatus* occurrence was sourced from *WildNet*, an electronic database managed by the Queensland Department of Environment and Heritage Protection [25]. The data collectively constitute observations from sources including the Australian Bird and Bat Banding Scheme, the Australian National Wildlife Collection, state museums, state wildlife atlases, and published and reputable unpublished research. Records span the period 1843–2007, with around 75% of the observations recorded since 1994, and cover all or part of the species mainland Australian distribution.

#### Environmental data

Spatial climate data (annual rainfall, annual minimum, maximum and mean temperature, relative humidity) for eastern Australia based on the most recent standard 30-year period 1961–1990 as defined by the World Meteorological Organisation [26], and normalised difference vegetation index (NDVI) for the recent peak HeV case years of 2011 and 2012 were sourced from the Australian Bureau of Meteorology [27]. Vegetation mapping (dominant species of the tallest stratum) was obtained from Geoscience Australia [28].

### Spatial Analysis

Analysis focused on eastern Australia, comprising the states of Queensland and New South Wales (where all reported equine Hendra virus cases have occurred) and the adjoining state of Victoria. Analysis on state-based boundaries (rather than biological boundaries) facilitated data collection. To facilitate computation, we used a 1% subset of the 118,900 potential control properties, as indicated above. We also merged the datasets of P. alecto and P. conspicillatus because of their demonstrated paraphyly [29] and evident species tropism of viral infections in bats [30]–[32]. All data were standardised by scaling from 0 and 1 to allow comparison of the coefficients [33].

Spatial autocorrelation (Global Moran’s I, zone of indifference conceptualisation) was used to identify spatial clustering and distances at which spatial processing was most pronounced [34]. Hot spot analysis (Getis-Ord Gi*, zone of indifference conceptualisation) identified areas with statistically significant clustering of Hendra virus spillovers [35]. Geographically weighted regression (GWS) was used to investigate the spatial pattern of spillovers in relation to environmental data. The latter were identified using ordinary least squares (OLS). Kernel density and inverse distance weighted were employed to interpolate data. All variables were included in the initial OLS model. A variable inflation factor (VIF) >7.5 suggested redundancy amongst variables and they were removed from the model in succession. Heteroscedasticity, or the relationship between predicted values and changes in variable magnitudes, was measured using the Koenker (BP) Statistic. OLS models that display statistically significant heteroscedasiticity (P<0.05) should use the reported Robust P, and are good candidates for GWR. Variables that did not have a statistically significant Robust P (P<0.1) were removed from the model in succession. Table 1 indicates the variables used in OLS and GWR. All spatial analysis was undertaken in Arcmap 10, Spatial Statistics Tools (ESRI, USA).

**Table 1:** Description of spatial and environmental variables used in OLS and GWR.

Variable	Description
P. alecto	Kernel density analysis of the recorded sightings of flying-foxes/km^2^
P. conspicillatus	“
P. poliocephalus	“
P. scapulatus	“
P. alecto/conspicillatus	Kernel density analysis of the merged recorded sightings of both *P. alecto* and *P. conspicillatus*/km^2^
Distance to roost	Distance to the nearest flying-fox roost
Horse population	Kernel density analysis of the population of horses/km^2^
Horse properties	Kernel density analysis of the number of horses properties/km^2^
Annual Rainfall	Average annual rainfall over the period 1961 to 1990
Annual Mean Temp.	Average annual mean temperature over the period 1961 to 1990
Annual Min. Temp.	Average annual minimum temperature over the period 1961 to 1990
Annual Max. Temp.	Average annual maximum temperature over the period 1961 to 1990
Relative Humidity 0900	Average relative humidity at 0900 hours over the period 1961 to 1990
Relative Humidity 1500	Average relative humidity at 1500 hours over the period 1961 to 1990
Vegetation	The dominant species of the tallest stratum

## Results

**Figure 1:**
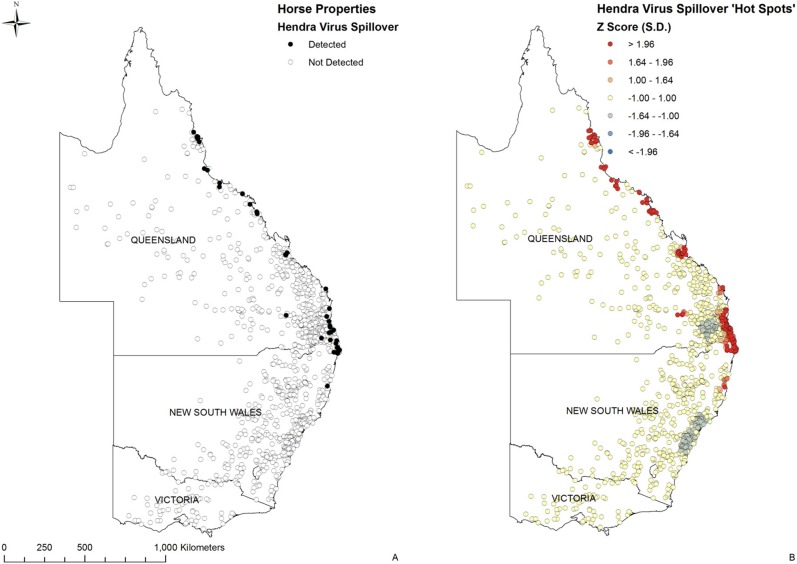
Equine property locations and Hendra virus spillover hot spots. (A) Forty reported Hendra virus equine cases September 1994 to December 2012 and 1,189 randomly selected control horse properties. (B) Hot spot analysis (Getis-Ord Gi*) identified areas of significant clustering of spill-overs (Z Score>1.96 SD) along the central and northern coasts of eastern Australia.

The 40 equine case properties reported up to December 2012 and the 1,189 randomly selected control horse properties are identified in Figure 1A.

**Figure 2:**
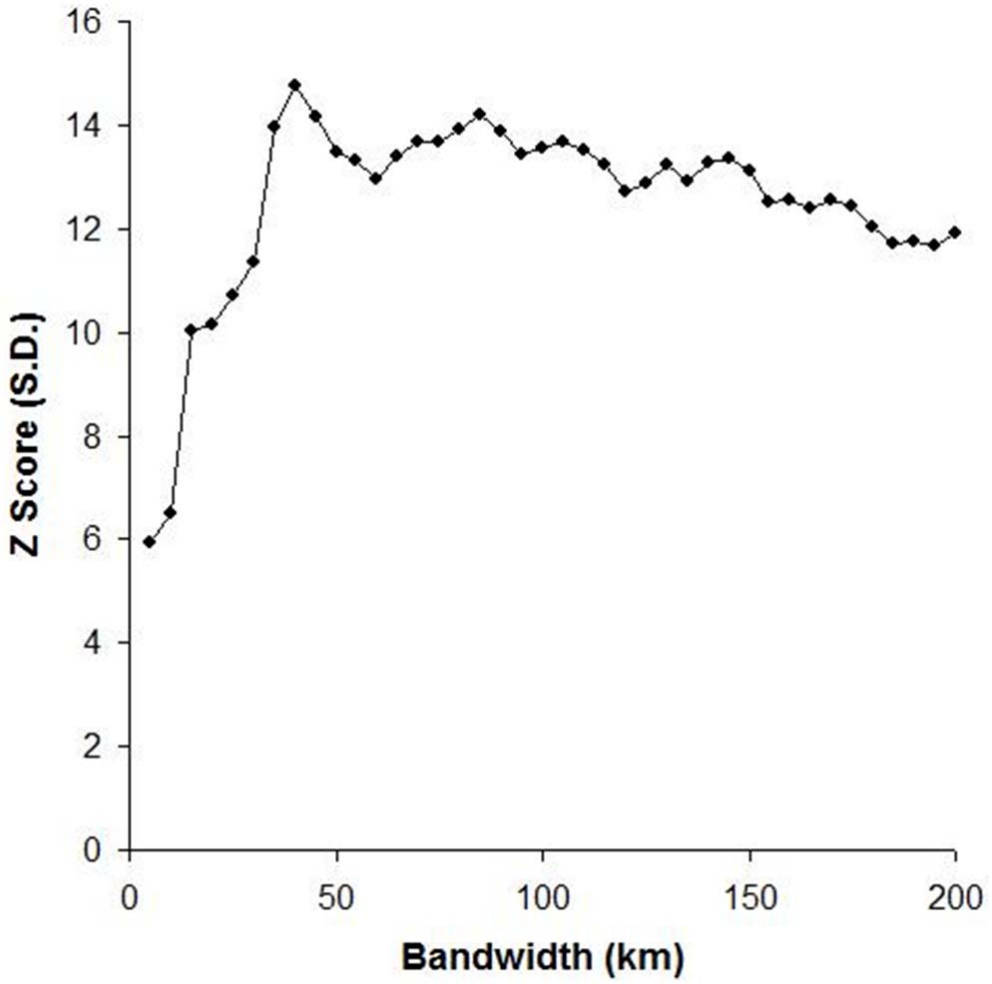
Spatial autocorrelation (Global Moran’s I) of Hendra virus spill-overs by distance. A peak Z Score at 40 km suggests that spatial processes exist at this distance to produce pronounced spatial clustering.

The Global Moran’s I test identified that spatial clustering of spill-over events was evident, and was statistically significant at a distance of 40 km (P<0.001) (Figure 2). This distance was then used as the ‘distance band’ in the Getis-Ord Gi* analysis (below) and as the search radius for interpolation.

Hot spot (Getis-Ord Gi*) analysis identified multiple areas of significant clustering along the east coast of Australia (Z Score >1.96 SD) (Figure 1B). Also interesting is the suggestion of ‘cold spots’, clustering of control properties on the Southern Downs of south-east Queensland and along the central coast of NSW (Z Score <1.00 SD).

**Figure 3:**
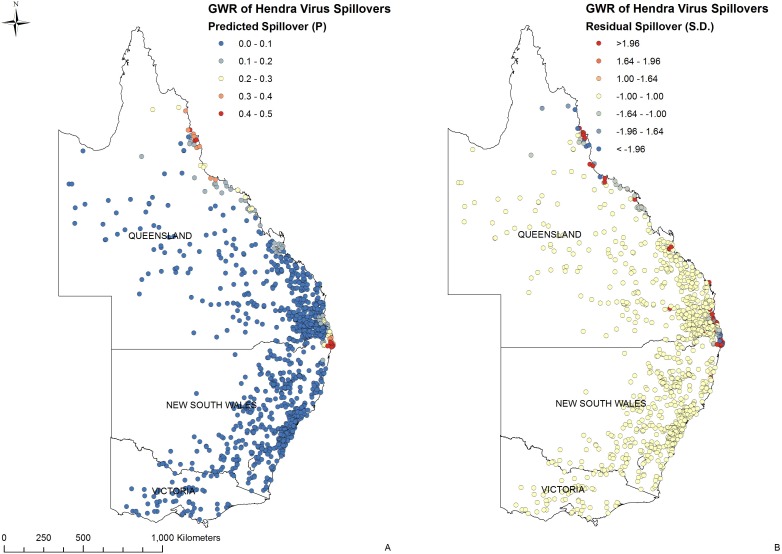
The final GWR model for the spill-over of Hendra virus in eastern Australia, with predicted (A) and residual (B) values. The density of flying-foxes *P. alecto* and *P. conspicillatus* had the strongest positive correlation with reported Hendra virus spill-overs (A). An absence of spatial autocorrelation of the residuals suggests additional (as yet unidentified) local risk factors play a role in Hendra virus spill-over from flying-foxes to horses (B).

Of the 15 spatial and environmental variables included in the study (Table 1), six were identified as being statistically significant in the initial and final OLS model (Table 2). Of these six, three had an association in the GWR, with the strength and direction of the correlation varying (Table 2). The density of flying-foxes *P. alecto* and *P. conspicillatus* had the strongest positive correlation (>0.5) with reported Hendra virus equine cases. The annual minimum temperature had a weak positive correlation (<0.2) and the density of *P. scapulatus* had a weak negative correlation (>−0.2). Negligible correlations (−0.1<0.1) were identified for *P. poliocephalus*, relative humidity (as measured at 0900) and horse population. The predicted (P) and residual (SD) values for a Hendra virus spill-over on horse properties are mapped in Figures 3A and B.

**Table 2:** Identification and modelling of spatial and environmental variables for Hendra virus spill-overs using OLS^a^ and GWR^b^.

	OLS Model (Initial)	OLS Model (Final)	GWR Model
P. alecto	0.57 (0.06)***		
P. conspicillatus	0.35 (0.07)*		
P. poliocephalus	−0.07 (0.05)*	−0.09 (0.04)***	−0.08 (0.08)
P. scapulatus	−0.21 (0.05)*	−0.23 (0.05)**	−0.20 (0.07)
P. alecto/conspicillatus	0.48 (0.06)***	0.53 (0.05)***	0.55 (0.06)
Distance to roost	−0.01 (0.05)		
Horse population	−0.09 (0.04)**	−0.07 (0.03)**	−0.05 (0.05)
Horse properties	0.01 (0.04)		
Annual Rainfall	0.02 (0.08)		
Annual Mean Temp.	0.20 (0.22)		
Annual Min. Temp.	−0.07 (0.16)	0.21 (0.03)***	0.14 (0.06)
Annual Max. Temp.	0.01 (0.16)		
Relative Humidity 0900	−0.06 (0.09)	0.14 (0.03)***	0.06 (0.06)
Relative Humidity 1500	0.21 (0.11)		
Vegetation	0.01 (0.01)		
Adjusted R^2^	0.18	0.18	0.21
AIC	−991.78	−994.07	−1037.00
Global Moran’s I (P)	0.53	0.18	0.62

a For the OLS models, estimates correspond to the standardised coefficient and the standard error in parentheses.

b For the GWR model, estimates correspond to the standardised mean coefficient and the standard error in parentheses.

*P<0.10,

**P<0.05,

***P<0.01 are statistically significant levels of the OLS model.

## Discussion

Hendra virus was initially perceived as a Queensland problem, with the first six reported equine incidents (1994–2004) occurring in this state. That perception changed in 2006 when the first equine case was reported in the neighbouring state of New South Wales. While it is probable that under-reporting of cases occurred historically, it is less likely post-2008, with increased industry and public awareness following the fatal infection of a veterinarian. However, any reporting bias is likely to be constant geographically, and thus unlikely to significantly impact our analysis. A lack of serological surveillance data precludes inclusion of sub-clinical infections. To date, all reported cases have occurred in eastern Queensland and New South Wales, within the variably overlapping geographic ranges of all four mainland Pteropus species [10]. We analysed the spatial distribution of reported Hendra virus infections in horses and identified that spatial clustering existed. Spatial clustering in the landscape is evidence of underlying spatial processes, and the pronounced clustering at 40 km, a distance consistent with the nocturnal foraging range of flying-foxes [36], [37] suggests the foraging ‘footprint’ of a roost as a biologically plausible underlying spatial dynamic.

Hot spot analysis (Getis-Ord GI*) is a useful tool to identify spatial clusters of both high and low values, and has previously been used to model disease occurrence [38]–[41]. Our analysis of Hendra virus spill-over events suggests multiple hot spots along the eastern Australia coast from far north Queensland to central New South Wales. The largest extends for nearly 300 km, and straddles the Queensland-New South Wales border (Fig. 1B). This hot spot includes almost half of all known events (19/40) to 31 December 2012. While it might be argued that the spatial significance is confounded by the unprecedented number of cases that occurred in 2011, when this year is excluded, this hot spot still accounts for nearly 30% of the events, and any effect of the temporal cluster would not fundamentally impact our analyses. From an epidemiological perspective, the preponderance of spillover events in this region suggests either the existence of a predisposing causal factor or better detection in this region. Combined, the identified hot spots contain about 7% of equine control properties, translating to around 8,000 equine properties at putative elevated risk of Hendra virus infection in eastern Australia, and suggesting advantage in spatially targeted risk mitigation approach.

In addition to identifying areas of evident increased spillover risk, the hot spot analysis also suggested areas of decreased risk, or ‘cold spots’. While the findings are not statistically significant, the putative ‘cold spots’ do correspond geographically to spatial clusters of equine control properties, and support the concept of a spatial risk gradient.

The geographically weighted regression analysis found the combined density of *P. alecto* and *P. conspicillatus* to be the strongest positive correlation with reported equine case locations in both the OLS and GWR models (Table 2). While the flying-fox dataset likely reflects observations of varying sampling intensity, the large number of observations and the extended timescale over which they were made supports its use to derive relative flying-fox species density. The spatial distribution of Hendra virus spill-over events and their association with flying-foxes has previously been reported [20], [42]. However, Field and Kung (2011) argue that the relationship between horses and flying-foxes alone does not explain the observed spatial pattern [43]. Field *et al* (2011) suggest that some species of flying-foxes may play a greater role in the transmission of infection to horses [44], implying that the pattern of spill-overs may better reflect species preponderance. This prospect is supported by the occurrence of multiple species of flying-fox on mainland Australia, by their differing geographic ranges, by the evident species-specific tropism of some bat viruses, and by the spatial occurrence of Hendra virus spill-overs. Our positive correlation of combined *P. alecto* and *P. conspicillatus* density with equine case locations supports this thinking, and suggests that these species play a primary role in the infection of horses. The frequent detection of Hendra virus genome in the urine of these two species further supports this contention [44] (Edson, D. *et al*, unpublished).

Roberts *et al* (2011) [24] reported the southward expansion of the range of *P. alecto*. Their reported rate of southern range expansion (100 km per decade) plus our finding of the strong correlation between *P. alecto/P.conspicillatus* density and equine case location, suggests the likelihood of a future southward expansion of Hendra virus equine cases, unless currently unidentified moderating factors exist. The absence of reported equine cases in NSW in the 1990s, the single case in 2006, and the cluster of cases in 2011 is consistent with this scenario.

The densities of *P. scapulatus* and *P. poliocephalus* have weak negative or negligible correlations with equine cases, reflecting the absence of equine cases in that part of their range not overlapping *P. alecto* or *P. conspicillatus*. This lack of positive correlation suggests *P. scapulatus* and *P. poliocephalus* play a limited role in the infection of horses, and is consistent with the infrequent detection of Hendra virus genome in the urine of these species [44] (Edson, D. *et al*, unpublished), despite the presence of neutralising antibodies to Hendra virus. While it might be suggested that the spatial correlation between equine case locations and the combined density of *P. alecto* and *P. conspicillatus* reflects an underlying latitudinal trend rather than a biological correlation with these two species, two observations argue against this: firstly, the absence of a positive gradient between far north Queensland and southeast Queensland (indeed the reverse is true); and secondly, the evident negative correlation between Hendra virus excretion in *P. scapulatus* and *P. alecto* at corresponding latitudes (Field, H. *et al*, unpublished).

Thus, an increasingly plausible scenario is the occurrence of species-specific Hendra virus ‘strains’ in Australian flying-foxes with varying infectivity and/or pathogenicity to horses. The related Nipah virus provides a relevant precedent, with the ‘Malaysian’ strain found in *P. vampyrus*, the ‘Bangladesh’ strain found in *P. giganteus*, and both strains detected in the geographically intermediate *P. lylei* in Thailand [45]. This thinking prompts science-based risk mitigation, and poses an alternative to the current approach of assessing risk on the basis of flying-fox occurrence collectively.

While the presence of horses is obviously a necessary component of equine case causality, we found that the density of horses had negligible correlation with equine case locations, and is evidently not a significant risk factor. McFarlane *et al* (2011) found the same [20]. This scenario plausibly reflects the ubiquitous presence of horses throughout eastern Australia, albeit at varying densities, and thus the *a priori* potential for spill-over. But given the presence of horses, our findings show that the primary spatial risk factor for spill-over and consequent equine cases is the occurrence of *P. alecto* or *P. conspicillatus.* However, the lack of clustering of the residuals in both the OLS and GWR models (Global Moran’s I, P = 0.32 and Figure 3B) suggests additional unidentified spatial risk factors likely play a role in Hendra virus spill-over. Visual inspection of the residuals indicates that case properties have a high positive residual (SD>1.96), while neighbouring properties have a negative residual (SD<–1.00). These findings suggest that while the distribution of *P. alecto* and *P. conspicillatus* is a significant global risk factor for spill-over, additional local risk factors play a significant, but as yet undetermined role. These factors might include property attributes, and husbandry and management practices that reduce the likelihood of flying-fox-horse interaction, such as excluding horses from paddocks where flying-foxes are active, and covering horse food and water points were possible [9]. Further investigations and comparisons between case and control properties are needed to confirm these local risk factors.

Our analysis of climate data sought to identify correlations consistent with the experimental findings of Fogarty *et al* (2008) who reported that Hendra virus survivability decreases dramatically as temperature increases [21]. Similarly, Scanlan *et al* (2014) modelled the effect of temperature on Hendra virus survival in the environment, finding a good temporal fit with the cumulative annual clustering of spill-overs in the Australian winter [46]. Our finding of a weak positive correlation (0.14±0.06) between a higher annual minimum temperature and spill-over is incongruent, and is likely confounded by the northern geographic distribution (higher minimum temperatures) of *P. alecto* and *P. conspicillatus*. More broadly, the absence of any identifiable climatic risk factor does not preclude its existence. It might be argued that the period of the current standard 30-yr climate dataset constrained our ability to identify any emerging climatic factors, but more fundamentally there are inherent challenges in terms of which variables to include in an analysis, and on what spatial scale. With a nomadic natural host species able to move hundreds of kilometres in a week, it is entirely plausible that a climatic risk factor might manifest remote to a subsequent spill-over location. Additionally, a climatic risk factor that acts indirectly and/or after a time-lag (for example, annual rainfall on subsequent food resource) poses confronting analytical complexity.

### Conclusion

The threat of Hendra virus causes particular anxiety and concern for horse owners in eastern Australia because of the very high case fatality rates in infected horses and close contact humans. Despite the recent availability of a vaccine for horses, it remains important that horse owners continue to implement risk management strategies that minimise horse exposure to Hendra virus. Our findings validate anecdotal assessments of a spatial risk component to Hendra virus infection in horses. They indicate that equine incidents are indeed spatially clustered, indicating underlying spatially occurring risk factors, and that multiple equine infection hot spots exist along the eastern Australia coast. Furthermore, we have shown that the density of two related species of flying-fox, *P. alecto* and *P. conspicillatus*, has a strong positive correlation with equine case locations. This finding suggests that these species play a primary role in Hendra virus infection of horses. We continue to seek other unidentified risk-contributing variables and to refine our model accordingly.

## References

[1] Murray K, Rogers R, Selvey L, Selleck P, Hyatt A, et al (1995) A novel Morbillivirus pneumonia of horses and its transmission to humans. Emerging Infectious Diseases 1: 31–33. 8903153 10.3201/eid0101.950107 PMC2626820

[2] Murray K, Selleck P, Hooper P, Hyatt A, Gould A, et al (1995) A Morbillivirus that caused fatal disease in horses and humans. Science 268: 94–97. 7701348 10.1126/science.7701348

[3] Selvey LA, Wells RM, McCormack JG, Ansford AJ, Murray K, et al (1995) Infection of Humans and Horses by a Newly Described Morbillivirus. Medical Journal of Australia 162: 642–645. 7603375 10.5694/j.1326-5377.1995.tb126050.x

[4] Baldock FC, Douglas IC, Halpin K, Field H, Young PL, et al (1996) Epidemiological investigations into the 1994 Equine Morbillivirus outbreaks in Queensland, Australia. Singapore Veterinary Journal 20: 57–61.

[5] Field H, Schaaf K, Kung N, Simon C, Waltisbuhl D, et al (2010) Hendra Virus Outbreak with Novel Clinical Features, Australia. Emerging Infectious Diseases 16: 338–340. 20113576 10.3201/eid1602.090780 PMC2958006

[6] Young PL, Halpin K, Selleck PW, Field H, Gravel JL, et al (1996) Serologic evidence for the presence in Pteropus bats of a paramyxovirus related to equine morbillivirus. Emerging Infectious Diseases 2: 239–40. 8903239 10.3201/eid0203.960315 PMC2626799

[7] Field HE, Breed AC, Shield J, Hedlefs RM, Pittard K, et al (2007) Epidemiological perspectives on Hendra virus infection in horses and flying foxes. Australian Veterinary Journal 85: 268–270. 17615038 10.1111/j.1751-0813.2007.00170.x

[8] Playford EG, McCall B, Smith G, Slinko V, Allen G, et al (2010) Human Hendra Virus Encephalitis Associated with Equine Outbreak, Australia, 2008. Emerging Infectious Diseases 16: 219–223. 20113550 10.3201/eid1602.090552 PMC2957996

[9] Anon. What is Hendra virus? The Dept of Agriculture, Forestry & Fisheries. Queensland Government, Brisbane, Australia. Available: http://www.daff.qld.gov.au/animal-industries/animal-health-and-diseases/a-z-list/hendra-virus/general-information/what-is-hendra-virus. Accessed: 26 Feb 2014.

[10] Field H, Crameri G, Kung N, Wang L (2012) Ecological aspects of Hendra virus. Curr Top Microbiol Immunol 359: 11–23. 22476530 10.1007/82_2012_214

[11] Young PL, Halpin K, Selleck PW, Field H, Gravel JL, et al (1996) Serologic evidence for the presence in pteropus bats of a paramyxovirus related to equine morbillivirus. Emerging Infectious Diseases 2: 239–240. 8903239 10.3201/eid0203.960315 PMC2626799

[12] Halpin K, Young P, Field H (1996) Identification of likely natural hosts for equine morbillivirus. Communicable Diseases Intelligence 20: 476.

[13] Halpin K, Young PL, Field HE, Mackenzie JS (2000) Isolation of Hendra virus from pteropid bats: a natural reservoir of Hendra virus. Journal of General Virology 81: 1927–1932. 10900029 10.1099/0022-1317-81-8-1927

[14] Field H, Young P, Yob JM, Mills J, Hall L, et al (2001) The natural history of Hendra and Nipah viruses. Microbes and Infection 3: 307–314. 11334748 10.1016/s1286-4579(01)01384-3

[15] Field H (2005) The ecology of Hendra virus and Australian bat lyssavirus. PhD thesis, The University of Queensland, Brisbane.

[16] Halpin K, Hyatt AD, Fogarty R, Middleton D, Bingham J, et al (2011) Pteropid Bats are Confirmed as the Reservoir Hosts of Henipaviruses: A Comprehensive Experimental Study of Virus Transmission. American Journal of Tropical Medicine and Hygiene 85: 946–951. 22049055 10.4269/ajtmh.2011.10-0567 PMC3205647

[17] Middleton D, Pallister J, Klein R, Feng YR, Haining J, et al (2014) Hendra virus vaccine, a one health approach to protecting horse, human, and environmental health. Emerging Infectious Disease. 20: 372–9 doi:10.3201/eid2003.131159 10.3201/eid2003.131159 PMC3944873 24572697

[18] Ostfeld R, Glass G, Keesing F (2005) Spatial epidemiology: an emerging (or re-emerging) discipline. Trends in Ecology & Evolution 20: 328–336. 16701389 10.1016/j.tree.2005.03.009

[19] Firestone S, Cogger N, Ward M, Toribio J-A, Moloney B, et al. (2012) The Influence of Meteorology on the Spread of Influenza: Survival Analysis of an Equine Influenza (A/H3N8) Outbreak. PLoS One 7. 10.1371/journal.pone.0035284 PMC3335077 22536366

[20] McFarlane R, Becker N, Field H (2011) Investigation of the Climatic and Environmental Context of Hendra Virus Spillover Events 1994–2010. Plos One 6. 10.1371/journal.pone.0028374 PMC3228733 22145039

[21] Fogarty R, Halpin K, Hyatt AD, Daszak P, Mungall BA (2008) Henipavirus susceptibility to environmental variables. Virus Research 132: 140–144. 18166242 10.1016/j.virusres.2007.11.010 PMC3610175

[22] Plowright RK, Field HE, Smith C, Divljan A, Palmer C, et al (2008) Reproduction and nutritional stress are risk factors for Hendra virus infection in little red flying foxes (Pteropus scapulatus). Proceedings of the Royal Society B-Biological Sciences 275: 861–869. 10.1098/rspb.2007.1260 PMC2596896 18198149

[23] Breed A, Breed M, Meers J, Field HE (2011) Evidence of Endemic Hendra Virus Infection in Flying-Foxes (Pteropus conspicillatus)-Implications for Disease Risk Management. PLoS One 6. 10.1371/journal.pone.0028816 PMC3237542 22194920

[24] Roberts BJ, Catterall CP, Eby P, Kanowski J (2011) Latitudinal range shifts in Australian flying-foxes: A re-evaluation. Austral Ecology 37: 12–22.

[25] Department of Environment & Heritage Protection. WildNet. Queensland Government, Brisbane, Australia. Available: http://www.ehp.qld.gov.au/wildlife/wildlife-online/index.html. Accessed: 26 Feb 2014.

[26] Bureau of Meteorology. Periods used in climate maps and statistics. Australian Government, Canberra, Australia. Available: http://www.bom.gov.au/climate/how/newproducts/map-periods.shtml. Accessed 5 May 2014.

[27] Bureau of Meteorology. Maps of average conditions. Australian Government, Canberra, Australia. Available: http://www.bom.gov.au/climate/averages/maps.shtml Accessed: 26 Feb 2014.

[28] Geoscience Australia. Vegetation-post-European settlement (1988). Australian Government, Canberra, Australia. Available: http://www.ga.gov.au/meta/ANZCW0703005426.html. Accessed: 26 Feb 2014.

[29] Almeida FC, Giannini NP, Simmons NB, Helgen KM (2014) Each flying fox on its own branch: A phylogenetic tree for *Pteropus* and related genera (Chiroptera: Pteropodidae). Mol Phylogenet Evol http://dx.doi.org/10.1016/j.ympev.2014.03.009. 10.1016/j.ympev.2014.03.009 24662680

[30] Nadin-Davis SA, Huang W, Armstrong J, Casey GA, Bahloul C, et al (2001) Antigenic and genetic divergence of rabies viruses from bat species indigenous to Canada. Virus Research 74: 139–156. 11226582 10.1016/s0168-1702(00)00259-8

[31] Chu DK, Poon LL, Chan KH, Chen H, Guan Y, et al (2006) Coronaviruses in bent-winged bats (Miniopterus spp.). J Gen Virol 87: 2461–2466. 16894183 10.1099/vir.0.82203-0

[32] Gloza-Rausch F, Ipsen A, Seebens A, Gottsche M, Panning M, et al (2008) Detection and Prevalence Patterns of Group I Coronaviruses in Bats, Northern Germany. Emerg Infect Dis 14: 626–631. 18400147 10.3201/eid1404.071439 PMC2570906

[33] BioMedware (2012) Methods for data standardisation. Avialable: http://www.biomedware.com/files/documentation/boundaryseer/Preparing_data/Methods_for_data_standardization.htm. Accessed: 26 Feb 2014.

[34] Li H, Calder C, Cressie N (2007) Beyond Moran’s I: Testing for Spatial Dependence Based on the Spatial Autoregressive Model. Geographical Analysis 36: 357–375.

[35] Getis A, Ord J (1992) The Analysis of Spatial Association by Use of Distance Statistics. Geographical Analysis 24.

[36] Markus N, Hall L (2004) Foraging behaviour of the black flying-fox (*Pteropus alecto*) in the urban landscape of Brisbane, Queensland. Wildlife Research 31: 345–355.

[37] Fox S (2011) The Spectacled Flying-fox-review of past and present knowledge. In: B. Law PE, D Lunney and L Lumsden, editor. The Biology and Conservation of Australasian Bats. Sydney, Australia.: Royal Zoological Society of NSW, Australia. 136–145.

[38] Kitron U, Kazmierczak J (1997) Spatial analysis of the distribution of Lyme disease in Wisconsin. Am J Epidemiol 145: 558–566. 9063347 10.1093/oxfordjournals.aje.a009145

[39] Brownstein J, Rosen H, Purdy D, Miller J, Merlino M, et al (2002) Spatial analysis of West Nile virus: rapid risk assessment of an introduced vector-borne zoonosis. Vector Borne Zoonotic Dis 2: 157–164. 12737545 10.1089/15303660260613729

[40] Martin V, Pfeiffer D, Zhou X, Xiao X, Prosser D, et al (2011) Spatial Distribution and Risk Factors of Highly Pathogenic Avian Influenza (HPAI) H5N1 in China. PLoS Pathog 7: e1001308. 21408202 10.1371/journal.ppat.1001308 PMC3048366

[41] Firestone S, Christley R, Ward M, Dhand N (2012) Adding the spatial dimension to the social network analysis of an epidemic: investigation of the 2007 outbreak of equine influenza in Australia. Prev Vet Med 106: 123–135. 22365721 10.1016/j.prevetmed.2012.01.020 PMC7126086

[42] Plowright R, Foley P, Eby P, Dobson A, Field H, et al (2011) Urban habituation, ecological connectivity and epidemic dampening: the emergence of Hendra virus from flying foxes (Pteropus spp.). Proceedings of The Royal Society B 278: 3703–3712. 21561971 10.1098/rspb.2011.0522 PMC3203503

[43] Field H, Kung N (2011) Henipaviruses-unanswered questions of lethal zoonoses. Current Opinion in Virology 1: 658–661. 22440924 10.1016/j.coviro.2011.10.025

[44] Field HE, De Jong C, Melville D, Smith C, Smith I, et al (2011) Hendra virus infection dynamics in Australian fruit bats. Plos One 6: e28678. 22174865 10.1371/journal.pone.0028678 PMC3235146

[45] Wacharapluesadee S, Boongird K, Wanghongsa S, Ratanasetyuth N, Supavonwong P, et al (2010) A longitudinal study of the prevalence of Nipah virus in Pteropus lylei bats in Thailand: evidence for seasonal preference in disease transmission. Vector Borne Zoonotic Dis 10: 183–90. 19402762 10.1089/vbz.2008.0105

[46] Scanlan J, Kung N, Selleck P, Field H (2014) Survival of Hendra virus in the environment-modelling the effect of temperature. EcoHealth DOI:10.1007/s10393-014-0920-4. 10.1007/s10393-014-0920-4 PMC7087565 24643861

